# Mechanical Transgressive Segregation and the Rapid Origin of Trophic Novelty

**DOI:** 10.1038/srep40306

**Published:** 2017-01-12

**Authors:** Roi Holzman, C. Darrin Hulsey

**Affiliations:** 1 Department of Zoology, Faculty of Life Sciences, Tel Aviv University, Tel Aviv 69978, Israel; 2 The Inter-University Institute for Marine Sciences, POB 469, Eilat 88103, Israel; 3 Department of Biological Sciences, University of Konstanz, Konstanz, 78457, Germany

## Abstract

Hybrid phenotypes are often intermediate between those of parental species. However, hybridization can generate novel phenotypes when traits are complex. For instance, even when the morphologies of individual musculo-skeletal components do not segregate outside the parental range in hybrid offspring, complex functional systems can exhibit emergent phenotypes whose mechanics exceed the parental values. To determine if transgression in mechanics could facilitate divergence during an adaptive radiation, we examined three functional systems in the trophic apparatus of Lake Malawi cichlid fishes. We conducted a simulation study of hybridization between species pairs whose morphology for three functional systems was empirically measured, to determine how the evolutionary divergence of parental species influences the frequency that hybridization could produce mechanics that transgress the parental range. Our simulations suggest that the complex mechanical systems of the cichlid trophic apparatus commonly exhibit greater transgression between more recently diverged cichlid species. Because (1) all three mechanical systems produce hybrids with transgressive mechanics in Lake Malawi cichlids, (2) hybridization is common, and (3) single hybrid crosses often recapitulate a substantial diversity of mechanics, we conclude that mechanical transgressive segregation could play an important role in the rapid accumulation of phenotypic variation in adaptive radiations.

Hybridization is often considered an impediment to the origin of phenotypic novelty. This is partly because over long evolutionary timescales, both genetic incompatibilities and locally advantageous genotypes will accumulate in isolated populations that result in hybrids being reproductively compromised and less locally adapted than parental species^1^,^2^,^3^,^4^. For example, the Dobzhansky-Muller (DM) model of hybrid incompatibility suggests that in two populations an ancestral genotype of AABB can evolve into AaBB in one population and AABb in another population. Although the new alleles could be neutral in their own genetic background, the novel combination of alleles a and b in a hybrid genotype AaBb could be inviable^5^,^6^,^7^. However, hybridization can also facilitate the origin of adaptive phenotypes. For instance, transgressive segregation is a phenomenon in which particular hybrid combinations at a mechanistic level of organismal design (e.g. genes) produce novel quantitative trait values (e.g. body size) outside the parental phenotypic distribution^8^,^9^. Empirical studies of transgressive segregation have generally led to an appreciation that hybridization can rapidly produce novel phenotypes through sorting of alternative alleles at the multiple loci underlying quantitative trait differences between species^10^,^11^,^12^,^13^ (Table 1). Based on a similar principal, transgression can also occur from novel combinations of skeletal components that contribute non-additively to the function of mechanical systems^14^,^15^ (Table 1). But, whether this mechanical transgression is a general characteristic of complex functional phenotypes and how it relates to the tempo of evolutionary divergence is not clear.

Functional abilities of the trophic apparatus that are critical to prey capture and handling are often emergent properties of multiple interacting musculo-skeletal components^16^,^17^,^18^,^19^. For example, in many vertebrates, bite force is a function of the lengths of both the lower jaw closing in-lever and out-lever that transmit force from the jaw muscles. In such composite mechanical systems, components can be altered in complex and non-additive ways to influence organismal function^17^. Because of the non-linear mapping of morphology to mechanics, slight alterations to morphology can result in disproportionately large functional changes^20^. Alternatively, divergent morphological configurations of the system can produce highly similar functional phenotypes, a widespread phenomenon in functional musculo-skeletal systems, known as “many-to-one mapping of form to function”^17^,^21^,^22^. This “many to one” complexity in the relationship between form and function has also been shown to have several important evolutionary consequences as it may (1) permit species to explore morphospace in non-linear ways^20^,^23^,^24^ and (2) reduce tradeoffs between components of multiple systems and thereby influence rates of evolution^19^,^25^. Additionally, morphological changes in the traits that underlie performance in composite mechanical systems can result in difficult to predict effects on the landscape of adaptive phenotypes^20^,^21^,^26^,^27^. The non-linearity of these functional systems might be especially important to an adaptive radiation when musculo-skeletal components of parental phenotypes are reshuffled during hybridization.

The mechanistic underpinnings of transgressive phenotypes are frequently not fully understood and therefore generalizing the results of transgression beyond the particular hybrids of two species is often challenging^28^. However, for phenotypes with explicit mechanistic models, we can simulate how hybridization should influence transgressive phenotypes across a large number of species^14^. In this context, radiations like the rapidly diverging clade of cichlids in Lake Malawi provide an ideal system to examine the potential role of transgressive mechanics during diversification. This is because there are several mechanical systems in the jaws of cichlids that are important during feeding, that are also characteristic of many other teleost species, and that can be explicitly modeled and compared to one another when their musculoskeletal components are measured^21^,^29^,^30^ (Fig. 1). Moreover, the vast mechanical and ecological diversity within this rapidly evolving clade permits the exploration of a broad trait space, providing robust inferences regarding the importance of transgression to adaptive radiation.

Additionally, the mechanics of the cichlid trophic system can be linked through functional morphology principals to their ecological consequences, permitting inferences of the functional significance of novel phenotypes. For instance, when capturing prey, fish rapidly expand their buccal cavity to generate a flow of water that pulls the prey into the mouth. The Suction Index^31^ summarizes suction feeding mechanics through the estimation of maximal buccal pressure as a function of force transmitted from the epaxial muscles as the cranium is elevated and the oral cavity is enlarged (Fig. 1B). Higher values of suction index are associated with greater flow speeds at the mouth^32^,^33^ that can be advantageous when removing attached prey, and lower values are associated with the ingestion of larger water volumes typical of predators that engulf bigger prey items like other fish^34^. Similarly, the anterior jaw four-bar linkage, that has been shown to exhibit transgression in cichlids^14^,^15^, is critical to both jaw protrusion and mouth closing^30^. This mechanical system transmits motion and force from the lower jaw to the maxilla through a ring-like configuration of skeletal elements (Fig. 1C). Mechanical modifications to this system that transmit more motion to the maxilla and produce greater jaw protrusion are associated with capturing evasive prey in cichlids^35^. Lastly, biting performance is another non-linear metric that is a direct result of forces transmitted from the adductor mandibulae muscle through the lower jaw closing lever system^29^,^36^ (Fig. 1D). Although most studies of the relationship of the lower jaw to feeding in fish have excluded the inputs from the muscles, more force modified lever mechanics of the lower jaw have consistently been shown to characterize species that specialize on attached prey or scrape algae^29^,^37^,^38^.

Mechanical transgression could be important to diversification during a radiation like the Lake Malawi cichlids for two reasons. First, transgressive segregation would be ecologically important if it allowed these organisms to rapidly invade a novel adaptive peak or to transition to another adaptive peak unoccupied by the parental species, reducing the competition between hybrid offspring and parental species. Second, mechanical transgressive segregation can be evolutionarily important in a rapidly diversifying clade, where genetic transgressive segregation is expected to have a limited role. This is because phenotypic transgression is generally thought to increase with the time of divergence between parental species^11^,^12^, However, it is not known if this pattern is altered in complex biomechanical systems, where individual components can recombine to produce highly non-additive mechanical phenotypes. Because recently diverged parental species could be mechanically similar, the variation created in hybrids of closely related species could frequently lie outside the parental range of mechanical phenotypes^15^. Alternatively, when the bones and muscles of two parental species recombine in morphologically divergent cichlid species that have not shared a common ancestor recently, a greater range of novel mechanical phenotypes could be generated^39^. It is unclear how divergence times (i.e. the time since the hybridizing species shared a common ancestor) and phenotypic complexity could interact to influence hybrid induced mechanical diversification during adaptive radiation.

To elucidate the potential for transgressive segregation to structure biomechanical divergence during adaptive radiation, we asked how the non-linear properties of three mechanical systems and the extent of parental evolutionary divergence interact to produce mechanical transgressive segregation in the Malawi cichlid trophic apparatus. We first generated a set of phylogenetic hypotheses to estimate relative divergence times among 35 cichlid species that together span a substantial component of the trophic disparity in Malawi cichlids. We then quantified the empirical divergence in the mechanical systems that characterize suction feeding, the anterior jaw four-bar linkage, and lower jaw closing force. Using this parameterized morphospace, we used simulations to ask when and to what extent can hybridization generate transgressive segregation in the mechanics of these functional systems. Within this framework, we focused on three questions: (1) Do the mechanical systems show differences in the degree of mechanical transgressive segregation? (2) How does the temporal divergence of hybridizing species influence the amount of mechanical transgression in the trophic apparatus? (3) Does mechanical transgression allow transitions between adaptive peaks unoccupied by the parental species?

## Results

### Properties of the mechanical systems across Malawi Cichlids

The suction index, describing the potential to generate low buccal pressure and corresponding fast suction flows, ranged from a low value of 0.05 in *Buccochromis heterotaenia* to a high of 0.30 in *Nimbochromis polystigma*. Several of the piscivorous species such as *Aristochromis christyi* and both *Tyrannochromis* species had low suction index indicating they likely ingest large volumes of water when engulfing fish. Benthically feeding species like *Nimbochromis linni, Tropheops microstoma,* and *Aulonocara stuartgranti* tended to have high suction index. The kinematic transmission coefficient of the upper jaw (hereafter “KT”), indicating the ability of the fish to rapidly extend their upper jaws to capture prey, ranged from 0.57 to 0.96, and the values are similar to those reported in Parnell *et al*.^14^ for Malawi cichlids. Several species that have little jaw protrusion and scrape algae like the *Metriaclima* and *Labeotropheus* species showed lower KT whereas some evasive prey specialists such as *Buccochromis heterotaenia* showed one of the highest KT values. The lower jaw closing force (F_close_), estimated based on the morphology of the jaw and its abductor muscles, ranged from 2.2 in the leaf-gleaning species *Hemitilapia oxyrhynchus* to 13.9 in the large-mouthed piscivore *Tyrannochromis macrostoma.* Other notable species that exhibited relatively weak F_close_ were the planktivores *Ctenopharynx pictus* and *Mchenga eucinostomus*. The fin biter *Genyochromis mento* showed relatively powerful F_close_ (10.2) especially when considered in the context of other closely related rock-dwelling cichlids that would be inferred to produce generally about one-third that bite force.

### Estimated phylogenetic relationships across Malawi Cichlids

The set of species trees we reconstructed to reflect the Malawi phylogeny (Fig. 2) was largely consistent with relationships recovered in Hulsey *et al*.^40^. In general, the deeper branches were well supported, while more recent divergence events generally had less support. Based on the posterior probabilities (% pp), there was substantial support for the splitting of the Malawi species examined into two major clades (≥99% pp). One of these clades contained what are generally thought of as mbuna rock-dwelling cichlids such as *Genyochromis mento* and *Melanochromis* spp., but the relationships within the mbuna did tend to exhibit low support values (≤50% pp). However, the other clade contained many species that are specialized to exploit the sandy habitats within Lake Malawi and included cichlids such as *Mchenga eucinostomus* and the *Tyrannochromis* species. This sand-dwelling clade was further divided into one clade (100% pp) containing the genera *Mchenga, Otopharynx, Ctenopharynx,* and *Cyrtocara* and another (100% pp) containing *Mylochromis mola* as sister to a larger clade that includes species ranging from Aristochromis christyi to the *Nimbochromis* species. A number of the relationships among species within this latter clade also showed substantial support (>50% pp).

### Mechanical Transgressive Segregation

Our simulations indicate that all Malawi species showed the potential for transgressive segregation in at least one of the mechanical systems (Fig. 2). However, a few species showed virtually no predicted transgressive segregation in one of the three systems. For instance, both *Tyrannochromis macrostoma* and *Taeniolethrinops praeorbitalis* would be predicted to exhibit virtually no transgressive segregation in the suction index when hybridizing with any Lake Malawi cichlid we examined. Similarly, no matter which species it hybridized with from Malawi, *Hemitilapia oxyrhynchus* would be predicted to exhibit very little transgression in F_close_ in its F2 progeny. However, generally all three systems and most species showed a propensity for mechanical transgression when forming F2 hybrids (Figs 2 and 3). The mean (±sd) proportion of hybrids that fell outside the parental range (“proportional expansion”) was 0.14 (±0.24) for the suction index, 0.34 (±0.32) for KT, and 0.12 (±0.24) for F_close_. The mean distance expansion was 1.85 (±7.1) for the suction index, 2.23 (±3.01) for KT, and 0.76 (±1.86) for F_close_ i.e. the range of mechanical transgression in the F2 generation was 0.76 times the range between the parental F_close_ values. On average, F2 hybrids between species pairs covered 32% (±17%) of the Malawi species range (“range expansion”) in the suction index, 56% (±34%) for KT, and 24 (±25%) for F_close_ (Fig. 3). While these simulations demonstrate that hybridization could produce individuals that fall outside the parental range, the vast majority (86%) of the offspring were well within the parental range.

Increasing the correlations between the traits that underlie performance generally decreased transgression. We simulated the suction index in F2 hybrids with increasing linkage strength, by adding (randomly) linkages between alleles, yielding an increasing degree of correlation (mean |r|) between the trait pairs of the suction index components (range 0.05–0.54). Transgression (measured as distance expansion) decreased significantly with increasing degree of correlation (spearman r = 0.23, p < 0.001).

The species values for the three transgression metrics were generally weakly correlated (Supplementary Table S1). There was some correlation between proportional expansion and distance expansion among hybridizing lineages (Mean Pearson’s *r* = 0.45 for the three systems). Large distance expansion in hybridizing species did not provide a clear predictor of range expansion (Mean Pearson’s *r* = 0.02 for the three systems). Species pairs that generated large proportional expansion often generated variable degrees of range expansion (Mean Pearson’s *r* = 0.07 for the three systems).

Overall, time had a marked effect on the patterns of transgressive segregation (Table 2). In two of the three functional systems (suction index and F_close_), the average transgression between the more closely related of the paired species was significantly higher than the null expectation. Suction Index showed significant negative correlation between cophenetic distance and proportional expansion and distance expansion, indicating higher transgression between more closely related species, and a marginally significant positive correlation (p = 0.053) with range expansion (Table 2). F_close_ showed significant negative correlation between cophenetic distance and proportional expansion and distance expansion and a positive significant correlation with range expansion (Table 2). KT values exhibited no significant effect of divergence time on transgression (Table 2). We attribute these trends to two patterns. First, across the three functional systems, parental species that had similar mechanical values (Suction Index, F_close_ or KT) showed higher proportional and distance expansion but lower range expansion (Fig. 4A,C,E). Second, Suction Index and F_close_ showed high to moderate degrees of phylogenetic signal (lambda; Table 2), meaning that more closely related species were more similar in their values. Thus, for these two functional systems, more closely related species would be more similar to one another, and would be characterized by higher proportional and distance expansion and lower range expansion. Note, however, that the strongest transgression occurred when the functional divergence was low to intermediate. For example, Fig. 4E indicates that the strongest transgression of KT values takes place at parental divergence of 0–0.2 compared to the full range of 0–0.4. Thus, mechanical transgression between species with similar functional traits can clearly contribute to generating functional diversity.

### Effects of Mechanical Transgressive Segregation on Adaptive Evolution

To evaluate the role of extreme phenotypes in generating transgression, we also calculated the degree of transgression as a function of the divergence between the parents (calculated as the maximal divergence) and the mean values in each functional system (Fig. 4B,D,F for KT values). Multiple regression analysis revealed that the degree of transgression was determined by the divergence between the parental pair (p < 0.05 for all three cases), with non-significant effect of the parent’s divergence between the mean of our 35 species (p > 0.01 for all three cases).

A SURFACE analysis conducted with the 35 parental species returned four putative evolutionary optima for this group of Lake Malawi species in 88 of 100 trees (Fig. 5A,B; 3 optima in the other 12 trees). The mbuna species that are primarily herbivorous tended to share similar mechanical optima likely because of their generally low KTs and low F_close_. The clade uniting *Cyrtocara taeniolatus* and *Placidochromis ornatus* often showed evidence of inhabiting their own unique mechanical optima relative to other sand-dwelling species like *Mchenga eucinostomus* and *Taeniolethrinops praeorbitalis.* In most reconstructions, the fin-biting species *Genyochromis mento* and the piscivore *Tyrannochromis macrostoma* shared a convergent mechanical regime that likely reflects their unusually high F_close_ coupled with unremarkable KT and Suction Index values. When F2 pairs where mapped on the SURFACE- generated optima, over 65% of the hybridizing pairs were found to produce offspring occupying at least one optima. Approximately 18% of all hybridizing pairs (104) generated offspring occupying an optima not previously occupied by either parental species. In 22 of the 595 pairs, two such optima (not occupied by the parental species) were occupied by at least one F2 from a single cross (Fig. 5C). While these results demonstrate that hybridization could produce individuals that fall on novel optima, it is important to note that the vast majority (96.5 ± 5.2%) of the offspring within each hybridization event fell outside any optima (including optima occupied by one of their parents).

## Discussion

### Implications for adaptive radiations

All three mechanically divergent trophic systems simulated here exhibited mechanical transgressive segregation, and this hybrid induced phenotypic divergence could play an important role in generating functional diversity during adaptive radiation for several reasons. First, all three mechanical systems in the Malawi cichlid trophic apparatus show substantial variability and, based on our simulations, could potentially produce transgressive mechanical phenotypes following hybridization (Fig. 2). Second, the phenotypic variation in hybrid trophic mechanics is often extensive. In some cases, the F2 phenotypes generated from individual hybridization events approximated the mechanical variation of the extant Malawi radiation (Figs 3 and 4). Additionally, these phenotypically far-reaching transgression events often enable a small minority of the F2 progeny to rapidly colonize evolutionary optima that were previously unoccupied by parental species (Fig. 5) and thus provide a putative mechanism to colonize optima that are outside the parental range. One of the hallmarks of adaptive radiation is the incredibly short timeframe over which their substantial phenotypic disparity has accumulated^40^,^41^,^42^,^43^,^44^, and mechanical transgressive segregation could be a mechanism that facilitates such rapid diversification (Fig. 5).

Our results highlight the non-intuitive observation that transgressive phenotypes do not always occur in crosses between phylogenetically divergent species (Figs 3 and 4). In fact, we found that hybrids of more recently diverged species often showed greater mechanical transgressive segregation as compared to hybrids of more evolutionarily diverged species. Although hybrids between more distantly related species could still produce exceedingly novel phenotypes as compared to more recently diverged species, mechanical transgression is clearly not just a phenomenon that should be considered as relevant to hybridization between distant relatives. The preponderance of greater mechanical transgression we found between more recently diverged species was especially strong for suction index (and for a lesser extent F_close_), and we attribute the degree of transgression to each system’s phylogenetic signature. For the three functional systems, parental species that had more similar mechanical values showed higher proportional and distance expansion but lower range expansion (Fig. 4A,C,E). However, this result does depend on the mode of trait evolution. If evolution erases the expected pattern of greater similarity in recently diverged species, the temporal signal of transgression could be lost. Yet, under the parameter range we explored, even low phylogenetic signal generated significant time-dependent transgression. This greater amount of transgression in recently diverged species starkly contrasts with the general empirical result that more temporally divergent species tend to show greater genetically fueled transgressive segregation^12^.

Critically, our models purposefully factored out morphological transgression. Therefore, we could not examine the degree to which transgression in morphology influences mechanical divergence relative to how only non-linear combinations of non-transgressive morphological components contribute to mechanical transgression. Any potential interaction between morphological and mechanical transgression could also be species- and system- specific and alter our inferences. However, less than 2% of individuals exhibited transgressive morphology in the one empirically generated hybrid cross that examined mechanical transgression^15^, indicating that such interaction might be infrequent.

In addition to the absence of morphological transgression from our model, there could be a number of reasons that we find instances of more transgression between more closely related species. First, lever-like musculo-skeletal systems as we examined here could have exceptionally non-linear relationships between their underlying components and emergent mechanics when compared to other physiological systems. Also, our mechanical systems could have the degree of complexity that incidentally is exceptionally favorable for transgression. A single component system would by definition have a linear relationship to mechanics while a system with tens of moving parts would be effectively reduced to an additive relationship between changes in component morphology and overlying function. Another possibility is that because researchers often believe transgression to be common only between very genetically distinct species, transgression might be less frequently studied when recently diverged species do hybridize and therefore transgression between these species is documented less frequently. More studies should explicitly examine transgressive segregation simultaneously in both recently diverged species and more genetically distinct lineages that can form hybrids.

Most studies examining hybrid transgression have focused on either qualitative differences between species or simple linear phenotypes that might only be expected to show transgressive segregation when species have diverged to the point that their hybrids exhibit substantially reduced viability as compared to parentals^11^. Even those studies that have examined multivariate phenotypes, have generally not been able to explicitly infer the mechanical or functional consequences of transgression (but see refs 3 and 4). However, our integrative analysis of all three functional systems enabled us to recover putative evolutionary optima from the mechanics of the parental species and assess the importance of hybridization in allowing F2 offspring to colonize optima not occupied by their parents. While most F2 within each hybridization event fall outside of these optima (i.e. in performance “valleys”, expected to result in compromised performance^3^,^4^) the few F2s that transgress to such optima would exhibit high performance. In the absence of competitors (when the radiation is young and species have not yet colonized these peaks), the rare F2s that fall on the optima will be theoretically able to colonize these new optima. While experiments with hybrids in other systems confirm that these beneficial F2s are rare^3^,^4^, they cannot preclude this scenario by virtue of their limited sampling. However, by focusing on functional traits that can be modeled biomechanically and that are known to be important in fish feeding, we were able to generate predictions that link the mechanical and performance landscapes on which fish evolve.

Comparisons of several metrics of transgressive segregation could generally serve to better delineate when transgression generates functional novelty. Because they are not highly correlated, our three transgression metrics could provide non-redundant inferences about the evolutionary importance of transgressive phenotypes. For instance, the generally large proportion of hybrid offspring that are transgressive for suction index inferred from the proportional expansion metric might provide information about hybrid competition with parental lineages^45^. Range expansion that quantifies the relatively small range of hybrid suction index phenotypes compared to other species in Malawi but come from the same parental crosses might indicate that hybrid offspring will show high overlap with other species in their utilization of prey requiring particular suction feeding abilities^27^. Furthermore, by calculating the range expansion (Table 2) we show that the mechanical variation produced during some simulated hybridization events approached or even exceeded the variation in all of the Lake Malawi species examined (Figs 3 and 4). Additionally, while transgression is essentially a relative trait (i.e. measured with respect to the parental range), comparing the offspring diversity to the range observed within the entire radiation provides a metric that is uniform across all species pairs within the radiation.

### Transgressive segregation and the rapid diversification of Malawi cichlids

Like many adaptive radiations, Lake Malawi cichlids exhibit a remarkable degree of trophic diversity including species that are specialized to feed on algae, fish parasites, mollusks, as well as the fins of other cichlids^46^. The inter-species variation in the complex functional systems that determine feeding performance is likewise considerable and comparable to that found in much older fish radiations^19^,^47^. Variation in these complex traits provide a critical prerequisite for our finding that virtually all of the Malawi cichlid lineages examined could, following hybridization, generate transgressive phenotypes (Fig. 2). By virtue of using three functional systems that differ in their numbers of components and non-linearity, we were able to explore a wide parameter space of mechanical possibilities.

Mechanical transgression has been shown to occur in a laboratory-raised hybrid cross of two closely related Malawi cichlids^15^. This cross resulted in over 50% of F2 individuals exhibiting transgressive phenotypes for four-bar linkage mechanics, while less than 2% of individuals exhibited transgressive morphology for any of the four components of the anterior jaw four bar linkage^15^. However, the amount of transgression observed in this cross might be unusual, because despite the four morphological components of the linkage differing in size between the two parental species, the anterior jaw KT values for the two parents were quite similar. Yet, this type of many-to-one mapping in mechanics whereby different morphological phenotypes can produce similar mechanical phenotypes is common in complex functional systems^20^,^22^,^24^,^26^,^48^, and mechanical transgression might be expected to most frequently occur when parental species exhibit little difference in emergent mechanical phenotypes.

Mechanical transgressive segregation might be particularly important in Lake Malawi cichlids. The incredibly recent timeframe during which the several hundred species within Lake Malawi have diverged has provided comparatively little time for intrinsic reproductive barriers to arise and reduce hybrid viability^15^,^49^,^50^. Therefore, hybridization and transgression in Lake Malawi cichlids could readily occur between two species with highly divergent trophic morphologies because hybridization between Lake Malawi cichlids is very common^11^,^14^. Indeed, ~1/4 of the species we included in the study have been confirmed to hybridize (Table S2). Additionally, because well over a hundred cichlid species can co-occur and breed in the same habitats, the opportunity for interspecific gene flow within this group of cichlids is exceptionally high^51^,^52^. This stands in contrast to many other classic adaptive radiations like Darwin’s Finches or *Anolis* lizards wherein it is relatively rare to find more than ten species together and most of these likely cannot form viable hybrids^53^,^54^. Additionally, a substantial diversity of the Lake Malawi radiation is made up of the rock-dwelling mbuna, cichlids that can be endemic to the rocky outcrops ringing individual islands^55^. Because water levels in Lake Malawi have changed extensively over time and repeatedly created new island habitats^56^, individual mbuna colonizing these islands might commonly be left with no reproductive option but to hybridize with other species. Therefore, the transient island-like habitat structure many Lake Malawi cichlids are confined to has likely favored hybridization and subsequent transgressive segregation to a much greater degree than larger and more stable habitats^52^,^57^,^58^. Furthermore, as opposed to the simple and relatively non-kinetic skulls of some successful groups like mammals or lizards^59^,^60^, the complex functional systems of the cichlid trophic apparatus exhibit the highly non-linear properties necessary for mechanical transgression^14^. The incredibly diverse cichlid flock in Lake Malawi might be especially prone to phenotypic divergence through mechanical transgressive segregation.

Features of complex systems such as many-to-one mapping and functional redundancy that likely contribute to the mechanical transgression explored here underlie a wide range of organismal functions^17^,^22^. Unraveling the importance of different levels of biological organization to the production of transgressive phenotypes will demand a more integrative view of when these particular mechanisms should contribute to transgression. Like the mechanical systems examined here, most functional systems are complex, vary quantitatively, and are composed of a number of components. Performance abilities as diverse as fish swimming^61^, damselfly escape performance^62^, shrew and bat bite force^63^,^64^, and turtle shell hydrodynamics^65^ are just a few of the complex phenotypes that are known to exhibit non-additive relationships between component structures, functional, and ecological adaptations. As our understanding of the mechanistic basis of organismal abilities increases, especially in organisms that are known to exhibit porous species boundaries, it will expand our ability to quantitatively assess the myriad functional consequences of hybridization.

## Methods

### Morphological Measurements

**Figure 1:**
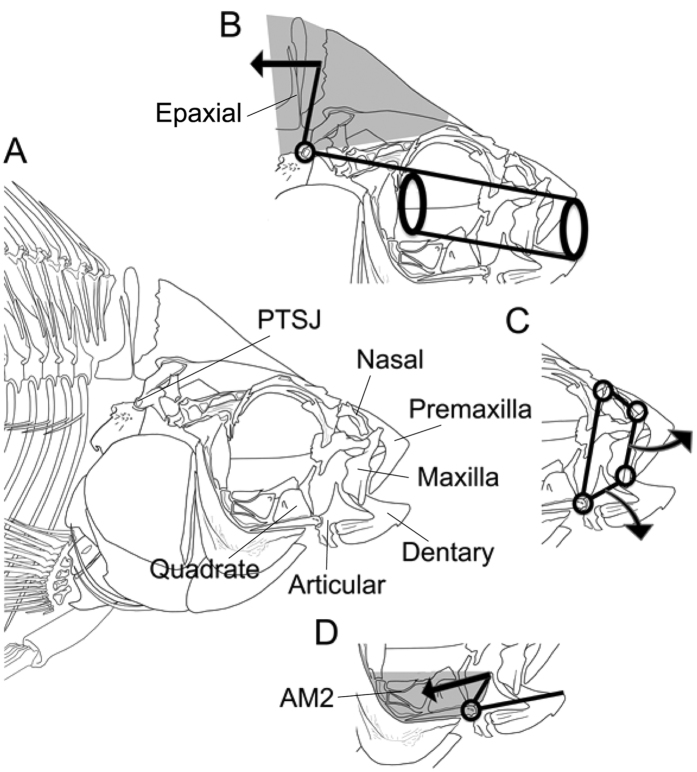
The morphology and mechanical systems examined in Lake Malawi cichlids for mechanical transgression. The skeleton of a generalized cichlid head is depicted (**A**) and several skeletal elements that contribute to trophic mechanics are labeled. In all of the systems, points of rotation are depicted with an open circle, the lengths of lever elements are shown with heavy black lines, the outline of muscles are shaded grey, and arrows highlight directionality of movement. The generalized mechanics of the suction index (**B**) are depicted. The suction index models the expanding cylinder of the buccal cavity as the epaxial muscle rotates the cranium posteriorly on the post-temporal supracleithrum-joint (PTSJ). Motion that is transmitted from the rotation of the lower jaw to the maxilla as it rotates to push open the premaxilla is represented with the anterior jaw four-bar linkage model (**C**). In the lower jaw closing system (**D**), the adductor mandibulae 2 (AM2) forcefully pulls the lower jaw closed across the quadrate-articular joint.

**Figure 2:**
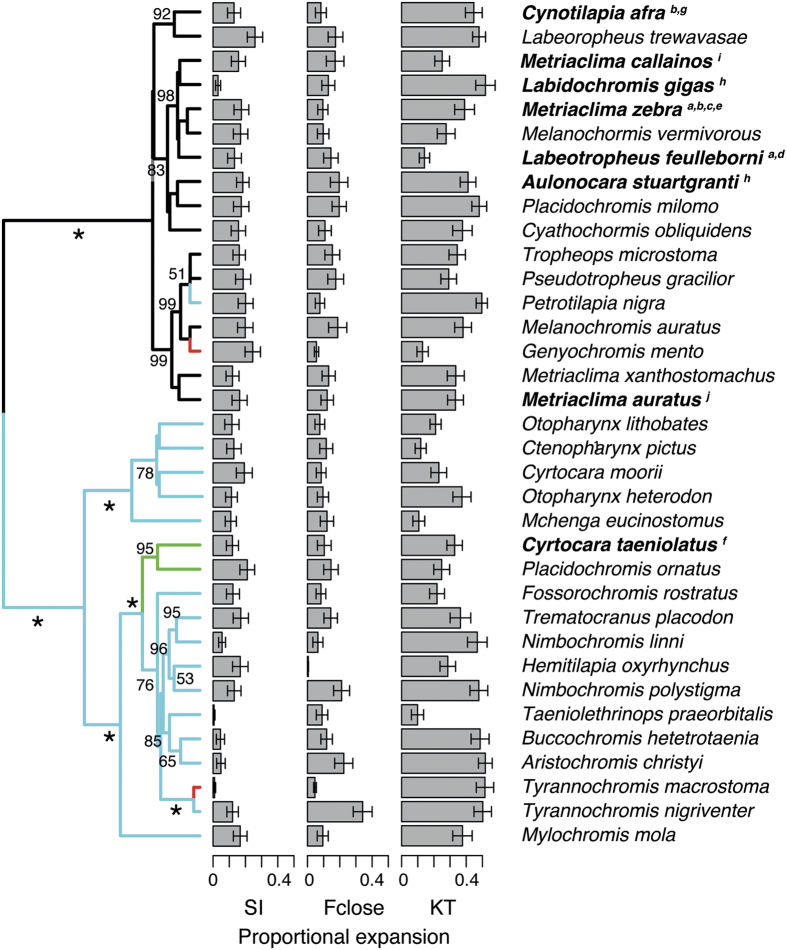
The inferred amount of transgressive segregation for the morphological potential to generate suction flows (suction feeding), the kinematic transmission coefficient of the anterior jaw four-bar linkage (KT), and lower jaw closing force (F_close_) were averaged for each species across all its simulated hybridization events and depicted on the consensus phylogeny inferred for the Malawi species. Eight species for which hybridization is confirmed appear in ***bold*** font, with superscript letters referring to references in Table S2. The ultrametric phylogenetic hypothesis shown above is the consensus tree of the post burn-in trees from five independent Bayesian analyses, each run for 5 million generations. The posterior probabilities above 50 for a given clade after averaging across the five runs are presented directly behind the node subtending each clade. Asterisks represent 100% posterior probability support for a node. The colors on the phylogeny represent four ‘Surface’ reconstructions of adaptive regimes within the Malawi cichlids. The size of the grey bars represents the mean proportional expansion for each species, and the inferred standard error across all possible hybridization events. Resampling for the hybridization analyses was repeated 10 times on each of the 100 topologies obtained from the combined BEAST runs.

**Figure 3:**
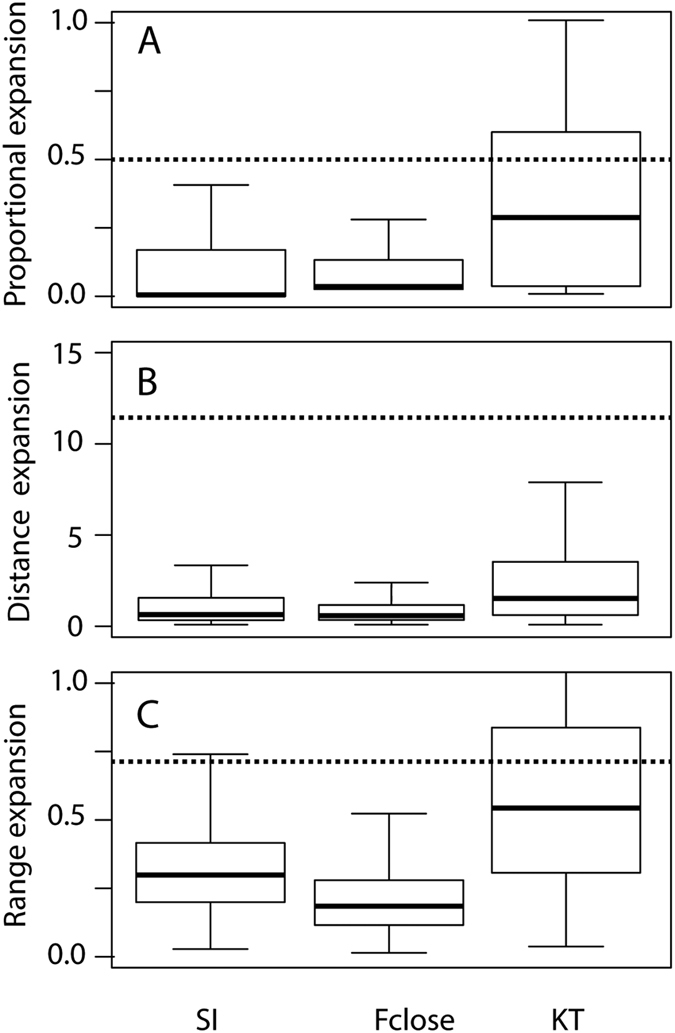
Transgression in the three functional systems we studied (Suction Index, F_close_, and KT) for simulated hybridizations between all possible pairs of 35 Malawi Cichlids (595 pairs). Three metrics of transgression are shown: (**A**) “proportional expansion”, defined as the proportion of F2 individuals with mechanical phenotypes outside the parental phenotypic range (**B**) “distance expansion”, defined as the ratio of the range of mechanical transgression in the F2 generation as a proportion of the range that the two parental mechanical phenotypes exhibited and (**C**) “range expansion”, defined as the ratio between the range of F2 mechanics and the range encompassed by our 35 divergent members of the Malawi cichlids. As a reference to the one empirically measured example, the dashed lines represent the degree of transgression reported in Parnell *et al*.^15^ for KT. Within each panel, horizontal bold lines indicate the median transgression; the upper and lower margins of boxes enclosing the mean indicate the 1st and 3rd quartiles, respectively; while whiskers represent 1.5 inter-quartile distances.

**Figure 4:**
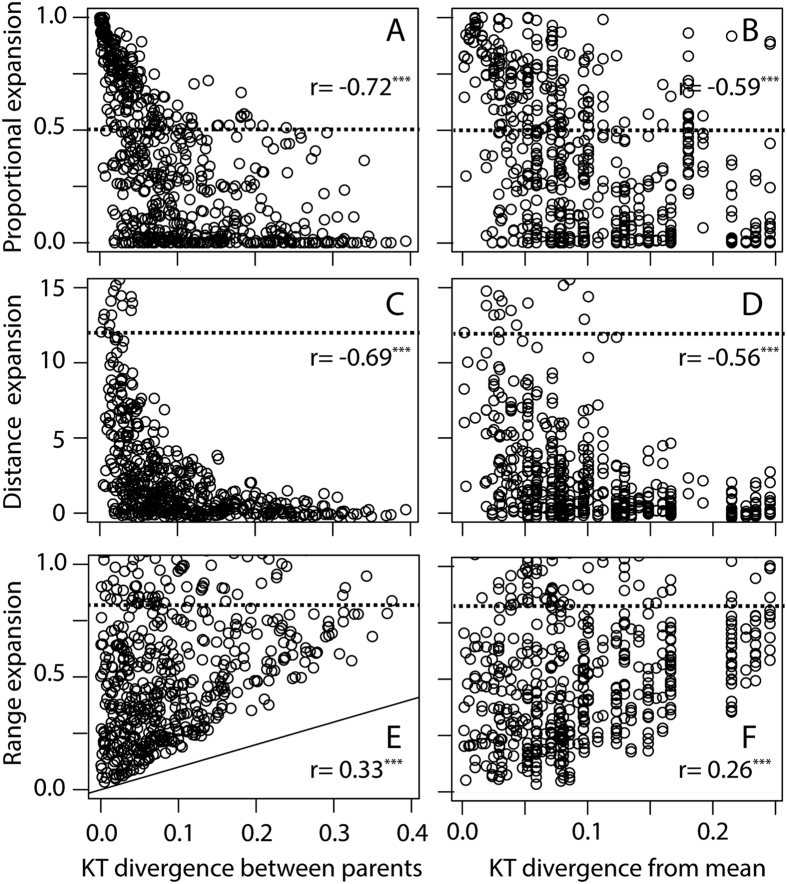
The degree of transgression (proportional, distance, range expansion; top to bottom panels) as a function of divergence in KT between hybridizing pairs (left row: **A,C,E**) and the divergence in KT between the parents and mean flock value (the largest of the two values; right row; **B,D,F**). The similarity in the mechanical properties of the functional system of the parents (**A,C,E**) was a better predictor of the degree of transgression, especially for distance and proportional expansion (**A,C**). The distinctiveness of the functional property (i.e. divergence of the KT of a species from the mean; **B,D,F**) was a weaker predictor of transgression. Results are not corrected for phylogenetic effects. The dashed lines represent the degree of transgression reported in Parnell *et al*.^15^ for KT. Diagonal full line in (**E**) is the degree of range expansion attributed to the distance between parents. Spearman r is indicated on each panel. n.s- non-significant, *p < 0.05, **p < 0.01, ***p < 0.001. Similar results were observed for the other two functional systems (not shown).

**Figure 5:**
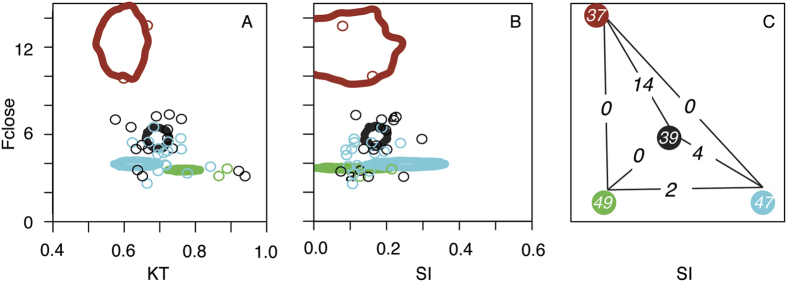
Mechanical transgressive segregation allows F2 hybrids to reach evolutionary optima not occupied by their parental species. A SURFACE analysis was used to estimate the location of evolutionary optima in the mechanical space defined by Suction Index, KT and F_close_. Circles represent the location of the 35 parental species, with colors correspond to their mapping in Fig. 2. A 2D representation of the 95% kernel around these estimates from our 100 trees is plotted in panel A (KT and F_close_) and B (Suction Index and F_close_). Using the simulation to produce 1000 hybrids from each of the 595 pair combinations, we asked how many optima not occupied by either parent are occupied by the F2s from a single hybridization event (**C**). Approximately 18% of all hybridizing pairs (104) generated offspring occupying one optimum not previously occupied by one of their parents (numbers in colored circles). In 22 of the 595 pairs, two such optima were occupied by at least one F2 (numbers on lines connecting circles). However, throughout our simulations, the vast majority (96.5 ± 5.2%) of the F2 offspring fell outside any optima (including optima occupied by one of their parents). The location of optima in (**C**) is not to scale.

We obtained measurements of the morphology and calculated the mechanics of three feeding-related functional systems: (1) the suction index (Fig. 1B), (2) the anterior jaw four-bar linkage kinematic transmission (KT; Fig. 1C), and (3) the lower jaw closing force (F_close_; Fig. 1D). The empirical morphological measurements were made on approximately three individuals of each Lake Malawi species. The specimens were obtained from the wild, formalin fixed, and preserved in 70% ethanol for long term storage. New measurements of all three systems were combined with measurements previously reported in a series of studies of Malawi cichlid trophic mechanics^14^,^40^,^66^. To obtain additional measurements, muscle masses were measured on a digital balance to the nearest 0.1 mg. Lengths of bony elements were measured to the nearest 0.1 mm with a dial caliper on specimens that were cleared using trypsin and stained with alcian blue cartilage stain and alizarin red bone stain^67^. To factor out the influence of size on our simulations of hybridization, we used the residuals from a log-log regression of standard length *versus* each component of the three focal functional systems to calculate the expected value of the component for a 100 mm standard length fish.

The three mechanical systems we parameterized using empirical measurements of the trophic apparatus have been repeatedly shown to capture the biomechanics underlying critical feeding abilities. The Suction Index summarizes the mechanical expansion of the buccal cavity in a suction-feeding fish during prey capture (Fig. 1B). The suction index is a metric that relates maximal buccal pressure to force transmission from the epaxial muscles as they elevate the cranium and expand the buccal cavity^31^. The suction index combines morphological measurements following the equation:


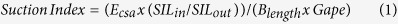


where *E*_*csa*_ is the epaxial muscle cross sectional area, *SIL*_*in*_ is the length of the moment arm for the epaxial muscles, *SIL*_*out*_ is the moment arm for the force due to the buccal pressure drop, and the area of the buccal cavity is the product of buccal length, *B*_*length*_, and gape width, *Gape*. The variable *Gape* is measured as 67% of the distance between the left and right coronoid processes of the mandible maximally opened, and to correspond to the time of peak buccal pressure. *B*_*length*_ is the distance between the anterior tip of the mandible and the posterior-most point of the basihyal. The length of *SIL*_*out*_ is taken as the distance between the post-temporal supracleithrum-joint (PTSJ) and the midpoint of the buccal cavity. *E*_*csa*_ is estimated from the area of a semi-ellipse that spans the PTSJ joint and the dorsal-most aspect of the epaxial muscle mass. Length of *SIL*_*in*_ is measured as the vertical distance between the centroid of the epaxial muscles and the PTSJ joint.

The anterior jaw (AJ) four-bar linkage is a lever system that translates lower jaw motion into upper jaw movement via the rotation of the maxilla and is directly involved in jaw protrusion that often reflects the amount of evasive prey in the diet^35^. There are four physical components of this four-bar linkage (Fig. 1C). The distance from where the nasal attaches to the neurocranium down to the coronoid process was measured as the fixed link (AJ_F_). As in all four-bar linkages, the fixed link is assumed to be immobile. The lower jaw rotates on this fixed link (AJ_LJ_) thereby serving as the input link that transmits motion into the system. The lower jaw link is measured from the base of the coronoid process, the joint where the articular rotates on the quadrate, to the ligamentous attachment of the maxilla on the dentary. We determined the distance between this attachment site of the maxilla on the dentary and the ligamentous connection of the nasal on the head of the maxilla. This was used as the mechanically relevant length of the maxilla (AJ_M_), and is the output link in the four-bar linkage that pushes the upper jaw open during jaw protrusion. The nasal bone serves as the coupler (AJ_N_) in this four bar linkage and is measured from where the nasal attaches to the neurocranium to where the bone ligamentously attaches to the maxilla. Using a series of trigonometric equations as reported previously for cichlids in Hulsey and García de León^35^, the kinematic transmission coefficient (KT) was calculated as the ratio of output rotation in the maxilla link (*Maxilla*_*angle*_) for a standardized input of 30 degrees of lower jaw rotation (*Lowerjaw*_*angle*_) as follows:


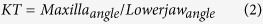


The force exerted during mouth closing (F_close_) is a critical function of the lower jaw^29^. In the lower jaw closing system, the adductor mandibulae 2 muscle actuates mouth closing and provides the primary force that is translated through the lower jaw closing lever system. The closing force F_close_ is determined by the following three component traits: (1) the cross sectional area of the adductor mandibulae 2 muscle (AM_csa_; estimated as the muscle mass raised to the 2/3 power), (2) the length of the moment arm for the AM2 muscle (LJ_in_), and (3) the moment arm for the force due to the closing jaw (LJ_out_) as follows:


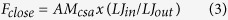


### Phylogenetic Reconstruction

To provide an evolutionary framework for our analyses, we estimated the phylogenetic relationships among 35 species and their putative outgroup *Rhamphochromis esox* that are all part of the Lake Malawi cichlid flock. We chose these species because they are generally common, frequently co-occur in Lake Malawi, include representatives of a large number of the named genera in Lake Malawi, and span a substantial amount of phenotypic diversity^46^. These species clearly do not represent all of the trophic diversity in the Lake Malawi cichlid flock, but we believe they provide a robust sample of the functional diversity within this assemblage. To generate an improved phylogenetic hypothesis for the relationships among the Malawi species examined, we utilized sequences that were reported in previous molecular studies of these species^40^,^42^. This allowed us to combine gene sequence data from five genetic partitions that included the mitochondrial nd2 and control region as well as the s7 intron 1, mitfb, and dlx2 nuclear partitions. Once parameterized, the five regions were combined to generate a distribution of phylogenetic hypotheses. The individual gene trees of Malawi cichlids likely show discordance due to the retention of ancestral polymorphism as well as hybridization^52^,^68^,^69^, Therefore, we used *BEAST 1.8^70^ to generate a species tree. For this tree, we ran five relaxed clock Bayesian analyses on the dataset partitioned by gene for 5 million generations with sampling every 5000 generations. At the end of each analysis, the log-likelihood scores were plotted against generation time to identify the point at which log likelihood values reached a stable equilibrium. In all five analyses, the equilibrium appeared to be reached at approximately 100,000 generations, and therefore, sample points prior to generation 500,000 in each run were discarded as “burn–in” samples. We also examined the effective sample size (ESS) of the likelihoods of the phylogeny remaining post-burn using Tracer v1.5^70^ to ensure that values were over 200, thereby confirming the phylogenetic search was relatively well mixed. From the five *BEAST runs, we isolated phylogenetic reconstructions evenly spaced through the tree search to generate a file with 100 phylogenetic trees. These phylogenies were used to provide a range of evolutionary hypotheses to run all of our simulations of hybrid phenotypic evolution, and thereby ensure that our results were not contingent on a single hypothesis of Lake Malawi cichlid relationships.

### Modeling the trophic apparatus in F2 Hybrid

To examine the propensity of our three complex functional systems to show transgressive segregation, we simulated 1000 hybrid F2 offspring between each pair-wise combination of the 35 Lake Malawi species in our cichlid phylogeny. This produced 595 simulated hybridizing pairs. Following the model outlined in Parnell *et al*.^14^, we constructed a simple individual-based Mendelian genetic model that assumes no mutation, no selection, and no error. Although these assumptions are not biologically realistic, this model assumes zero phenotypic variance in the parental species and makes modeling and comparing F2 hybrid phenotypes tractable. In the model, we posited that four independent loci with two alleles at each locus determined the size-standardized length and cross sectional area of each of the components for each F2 individual. This gave us eight alleles per component. The parental species with the largest empirical values for a component was treated as fixed for all large alleles and the parental species with the smallest component value was treated as fixed for all small alleles. The effect of each allele was assumed to be equal, additive, and size-independent. Therefore, these eight alleles allowed us to divide the size differences of each component (for example, nasal link length in the four bar linkage; Fig. 1) between the two hybridizing parents into eight segregating genotypic units that had equal influences on their F2 hybrid nasal length differences.

In our dataset, components of the functional systems showed varying degrees of correlations (0.01 < |r| < 0.78; Supplementary Figs S1–S3) when analyzed using independent contrasts across the phylogenetic hypotheses of the Lake Malawi cichlids. Therefore, we modeled the genetic correlation between components within each functional system. To do this, we linked the simulated inheritance of alleles within pairs of components of each system to achieve correlations that were statistically indistinguishable from the phylogenetic correlations (mantel test, P > 0.2 for all cases). The particular alleles that were linked were determined arbitrarily. The same degree of linkage was used for all the F2 crosses. This procedure ensured that the modeled size of a component (e.g. nasal length) would follow the observed correlation with the size of the other components (e.g. maxilla length). In some crosses, a small number (<5 simulated individuals) of the hybrids produced invalid four bar geometries. These simulated individuals were removed from the dataset and re-sampling was conducted to reach 1000 individuals. While this model makes several simplifying, unrealistic assumptions, it is designed to capture the genetic mechanism underlying the determination of morphological traits as we understand them. For example, the approximate number of alleles per trait, their relatively equal effect size, and the fact that some alleles are linked while some are not, are based on QTL studies within the functional systems we are modeling^11^,^15^,^36^,^71^. Furthermore, this model was shown to be consistent with hybridization experiments in our study system^15^.

The model purposefully excluded the possibility of morphological transgression in all of the hybrid crosses. Effectively, the additivity and distribution of allelic effects ensured that all F2 morphological phenotypes could only fall within the range of parental morphologies. Critically, if transgressive segregation of mechanical phenotypes were recovered in the F2 generation, this should only result from non-linear mapping of component morphological phenotypes onto inferred hybrid F2 mechanics. Empirical quantitative genetic data from a number of studies of the cichlid craniofacial skeleton suggests that our assumptions of number of loci per component as well as the additivity of allelic effects influencing component size are generally realistic^11^,^15^. Precluding any transgressive segregation in the morphology of a hybrid is also consistent with empirical findings in Lake Malawi cichlids reported in Parnell *et al*.^15^.

### Comparative Analyses of Transgressive Segregation

Trait phenotypes can quantitatively differ in how they transgress parental values, and how one quantifies transgression could lead to different inferences concerning transgression’s evolutionary importance. For instance, the proportion of hybrid offspring that exhibit transgressive phenotypes could readily influence hybrid viability during competition with parental lineages^45^. Similarly, the range of mechanical phenotypes produced via hybridization might influence both the potential for functional novelty and simultaneously capture the expected amount of niche overlap among hybrid siblings^72^. It has also recently been demonstrated empirically that a hybrid cross between two closely related cichlid species produced offspring with mechanics approaching the variation observed across a substantial diversity of Malawi cichlids^15^. This hybrid-induced exploration of the mechanical landscape within only a few generations could determine how transgression in particular functional systems might influence rates of phenotypic evolution^39^. Therefore, comparing several metrics of transgressive segregation could allow us to more clearly understand the evolutionary consequences of particular hybridization events as well as when transgressive segregation should most likely generate functional novelty.

Following our simulations of mechanical transgression for the three mechanical systems in each of the 595 crosses of Malawi species, we calculated three metrics of transgressive segregation for the 1000 F2. First, we determined the proportion of F2 individuals with mechanical phenotypes outside the parental phenotypic range (hereafter “proportional expansion”). For example, if 50 of the 1000 simulated F2 individuals from a particular hybrid cross were found to exhibit mechanical phenotypes outside the parental range, the proportional expansion would be 0.05, or 50/1000. Then, we calculated the ratio of the range of mechanical transgression in the F2 generation as a proportion of the range that the two parental mechanical phenotypes exhibited (hereafter “distance expansion”). For example, if the range of F2 values for KT were 0.50 to 1.00 and the parental species values were 0.70 to 0.95 then the F2 would transgress the smaller parental value by 0.20 and the larger parental value by 0.05. The distance expansion would therefore be 1.0, or (0.20 + 0.05)/(0.95 − 0.70), and the F2 would encompass the parents’ mechanical range as well as a novel distribution of mechanical phenotypes equal to the distance between the two parents. Additionally, we determined the ratio between the range of F2 mechanics and the range encompassed by our 35 divergent members of the Malawi cichlids (hereafter “range expansion”). If the range of F2 values for KT was 0.6 to 0.8 (0.2) and all 35 Malawi cichlid species had a range between 0.56 to 0.96 (0.4) then range expansion was 0.5; i.e the F2 hybrids occupy half of the range of the range of all 35 Malawi cichlid species. These three metrics were compared among the three functional systems. Note that while “proportional expansion” and “distance expansion” are measures of transgression that are made relative to the parental species, the metric “range expansion” is calculated not with reference to parental values, but in reference to the entire range exhibited by the 35 Malawi species.

To determine whether the three metrics provided independent information on the tendency of species to show mechanical transgression, we examined the relationships between species average values of the three metrics using a Mantel test. For each of the three mechanical systems, we calculated Pearson correlation coefficients (*r*) of the pair-wise species average relationships between proportional expansion, distance expansion, and outside Malawi range. Although the potential for a species to show transgressive segregation is not solely due to its own phylogenetic history, we also examined species average values between pairs of the three metrics using independent contrasts and found similar results to the Mantel tests (results not shown).

We also examined how temporal divergence played a role in the extent of mechanical transgression. For each phylogenetic tree, we first calculated the three metrics (i.e. proportional, distance and range expansion) for the 595 species pairs. We then noted the spearman correlation coefficient between each metric and the cophenetic distance between the simulated parental species. We tested whether this temporal pattern was significant using a randomization procedure, by calculating the spearman correlation after reshuffling cophenetic distances between the 595 parental pairs. For each phylogenetic tree, we repeated the reshuffling 100 times, and the number of times in which the randomized spearman correlation was higher (or lower) than the observed one. This procedure was repeated for each of 100 phylogenetic trees. Significance level was calculated by dividing the total number of times in which the randomized spearman correlation was higher (or lower) than the observed one, divided by the number of simulations (100 phylogenetic trees × 100 simulations).

Finally, we asked if mechanical transgression would allow hybrids of Lake Malawi cichlids to invade novel peaks in the adaptive landscape. To test this idea, we used the CRAN package Surface in R^73^ to reconstruct the inferred evolutionary optima for the three mechanical performance metrics (Suction Index, KT, and F_close_) based on the 35 parental species of Lake Malawi cichlids we examined. Surface uses stepwise AIC algorithms to generate a series of ‘Hansen’ models that estimate convergent regimes within a clade. This procedure identified 4 putative optima in 88 of the 100 trees, and 3 optima in the other 12 trees (see Results). We then estimated the locations of these evolutionary optima in the mechanical space using inferred locations from our 100 trees to calculate the 95% density estimate for the 3 dimensional regions. The calculated optima represented relatively small volumes of mechanical space abstracted from the data set of our 35 species, and none of the individual species were inferred to inhabit these volumes. Therefore, our analysis provided conservative estimates of the size of optima in the biomechanical landscape as well as the probability of hybrid offspring occupying the optima. Following the reconstruction of the optima, we assigned parental species to the closest optima. We then asked, for the 1000 simulated F2 hybrids from each cross, how many novel evolutionary optima (not occupied by neither parent) were occupied by at least one hybrid offspring.

## Additional Information

**How to cite this article**: Holzman, R. and Hulsey, C. D. Mechanical Transgressive Segregation and the Rapid Origin of Trophic Novelty. *Sci. Rep.* **7**, 40306; doi: 10.1038/srep40306 (2017).

**Publisher's note:** Springer Nature remains neutral with regard to jurisdictional claims in published maps and institutional affiliations.

## Supplementary Material

Supplementary Figures and Tables

## Figures and Tables

**Table 1:** Morphological and mechanical transgressive segregation through hybridization.

Genetic transgressive segregation				Mechanical transgressive segregation			
QTL	P1	P2	Example hybrid F2s	Component	P1	P2	Example hybrid F2s
Locus 1	−1	+1	−1	Muscle force	2.0	6.0	4.0
Locus 2	+1	−1	−1	Out-lever	2.0	3.0	2.5
Locus 3	−1	+1	−1	In-lever	2.0	1.0	1.5
Phenotype	−1	+1	−3	Closing force	2.0	2.0	2.4

Genetic transgression can arise when a trait is determined by multiple loci, with each loci being fixed for alternative alleles to a different value (+1 or −1) in two parental populations P1 and P2. If these two species hybridize, the F1 progeny will be heterozygous for the hypothetical trait, and the F2 progeny will show all the possible genotypes from a homozygous +1 (with an overall value of +3) to a homozygous −1 (with an overall value of −3), thus transgressing the parental range of −1 to +1. Based on a similar principal, mechanical transgression can arise when a trait is determined by multiple components (e.g. the lower jaw closing lever; Fig. 1; see equation 3 in *Methods*), fixed to different values in two parental populations P1 and P2. In the F2 hybrids, each component would have a value that is within the range of the two parental values (the mean in this hypothetical example). Thus, no genetic transgression is assumed. However, because in complex functional systems the relationships between the components and performance are often non-linear, the emerging trait value (closing force) can transgress the parental range.

**Table 2:** Temporal pattern in transgressive segregation in lake Malawi Cichlids. r is spearman correlation coefficient between the degree of transgression (three different matrixes) and divergence times between each pair of transgressed species (averaged across 100 trees).

Trait	Prop exp	Dist exp	Range exp	Lambda
Suction Index	r = −0.20 p = 2e–4***	r = −0.20 p = 2e–4***	r = 0.09 p = 0.053^@^	0.53 ± 0.26
F_close_	r = −0.23 p < 1e–4***	r = −0.23 p < 1e–4***	r = 0.12 p = 0.013*	0.18 ± 0.05
KT	r = 0.016 p = 0.61^ns^	r = −0.001 p = 0.49^ns^	r = 0.06 p = 0.12^ns^	0.015 ± 0.002

Significance was assessed through a randomization test that shuffled species on the tree (n = 100 randomizations per each of 100 trees). Lambda, the degree to which phylogeny predicts covariance among trait values for species, was averaged (±sd) for the 100 trees. Prop exp- proportional expansion; Dist exp- distance expansion; Range exp- range expansion. n.s- non-significant, @-marginally significant, ^*^p < 0.05, ^***^p < 0.001.
