# Supplementary material for: Mechanical Transgressive Segregation and the Rapid Origin of Trophic Novelty
Source: Sci Rep. 2017 Jan 12;7:40306. doi: 10.1038/srep40306 (PMC5228120; doi:10.1038/srep40306)
Supplement: Supplementary Figures and Tables [file srep40306-s1.pdf]

Supplementary materials for: Mechanical Transgressive Segregation and the Rapid Origin of Trophic Novelty

Authosr: Roi Holzman[1,2,*], C. Darrin Hulsey [3]

[1] Department of Zoology, Faculty of Life Sciences, Tel Aviv University, Tel Aviv 69978, Israel.

[2] The Inter-University Institute for Marine Sciences, POB 469, Eilat 88103, Israel.

[3] Department of Biological Sciences, University of Konstanz, Konstanz, Germany, 78457.

* corresponding author

Running title: Tempo of Hybrid Jaw Mechanics

Fig S1: Phylogenetic-corrected correlations between components of the suction index. Statistically significant correlations appear in **bold** font. Pearson correlation coefficients (r) are averaged across the 100 phylogenetic trees used in the study, and the average value was used to calculate the p-values. Data presented in the figure are independent contrasts from an arbitrarily selected phylogenetic tree. E_{csa} is the epaxial muscle cross sectional area, SIL_{in} is the length of the moment arm for the epaxial muscles, SIL_{out} is the moment arm for the force due to the buccal pressure drop, and the area of the buccal cavity is the product of buccal length, B_{length}, and gape width, *Gape*.

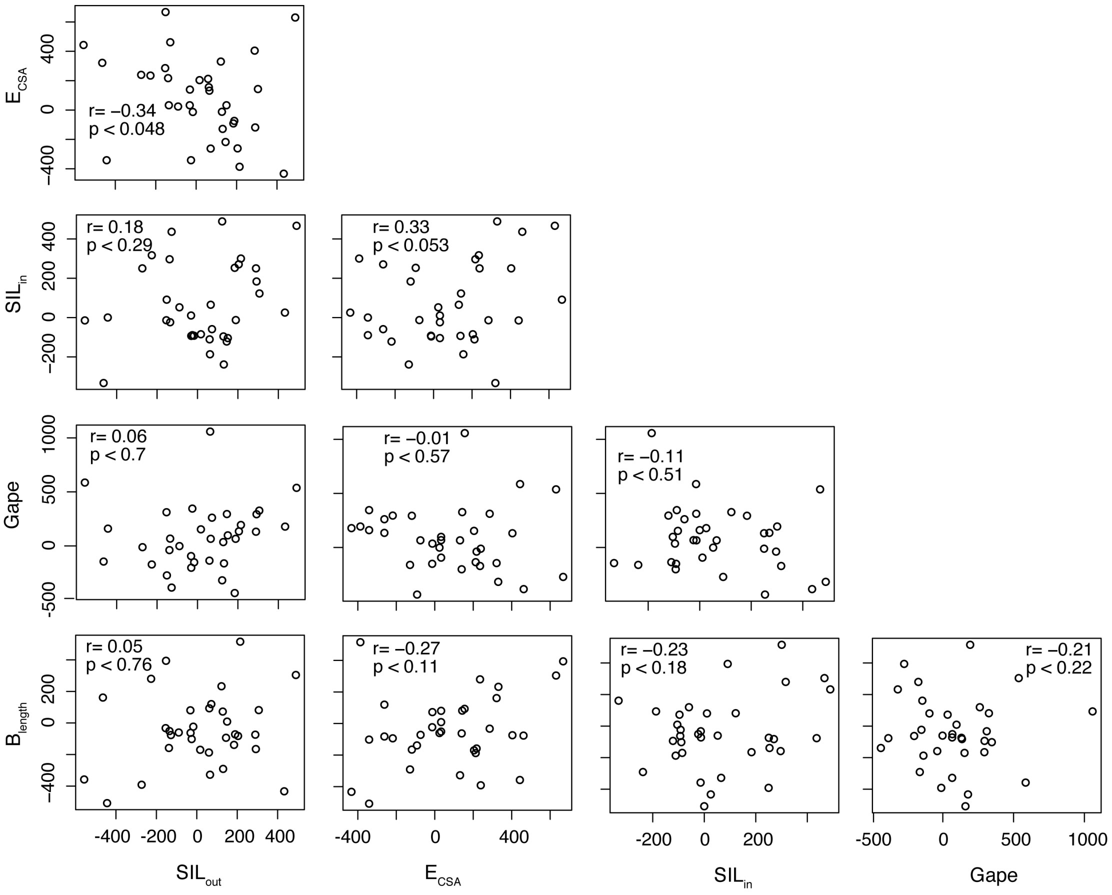

Fig S2: Phylogenetic-corrected correlations between components of the lower jaw closing system used to calculate F_{close}. Pearson correlation coefficients (r) are averaged across the 100 phylogenetic trees used in the study, and the average r value was used to calculate the p-values. Data presented in the figure are independent contrasts from an arbitrarily selected phylogenetic tree. AM_{csa} is the cross sectional area of the adductor mandibulae 2 muscle, LJ_{in} is the length of the moment arm for the AM2 muscle and LJ_{out} is the moment arm for the force due to the closing jaw.

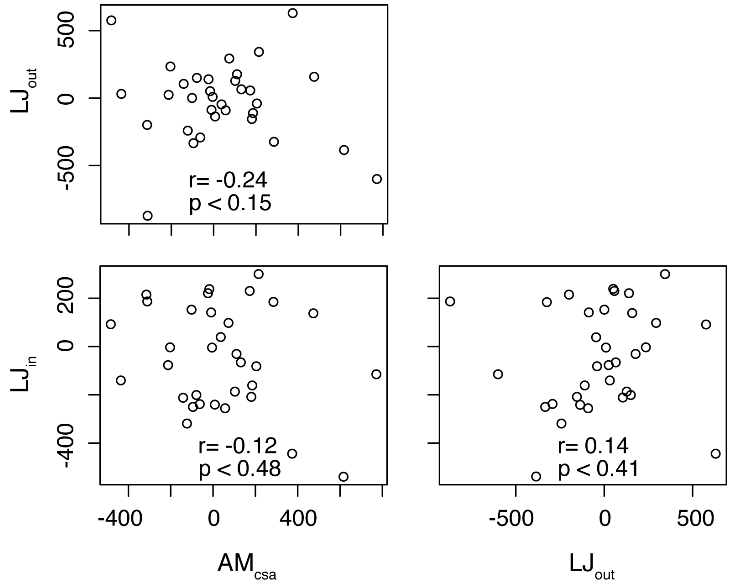

Fig S3: Phylogenetic-corrected correlations between components of the anterior jaw four-bar linkage system used to calculate KT. Pearson correlation coefficients (r) are averaged across the 100 phylogenetic trees used in the study, and the average r value was used to calculate the p-values. Data presented in the figure are independent contrasts from an arbitrarily selected phylogenetic tree. AJ_F - fixed link, AJ_{LJ} - lower jaw link, AJ_M – maxillary link, AJ_N – nasal link (coupler).

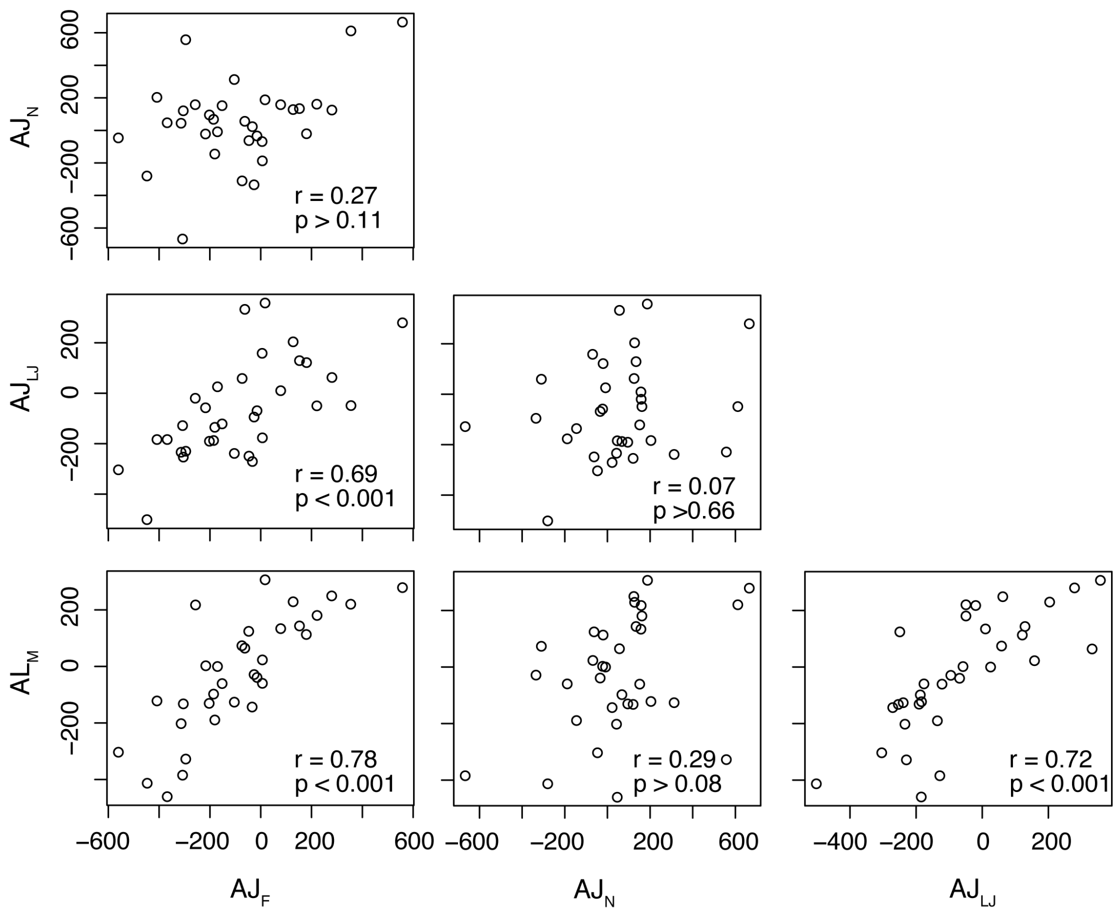

Table S1: The correlations among our three transgression metrics for the three mechanical systems. We used a Mantel test to calculate correlation coefficients, r, and associated p-values, between cichlid species values of proportional expansion, distance expansion, and outside the Malawi range for suction index (SI), the kinematic transmission coefficient of anterior jaw four-bar linkage system (KT), and the lower jaw closing system (F_{close}).

System	Proportional Expansion vs. Distance Expansion	Distance Expansion vs. Outside Malawi Range	Proportional Expansion vs. Outside Malawi Range
SI	0.67 ($P < 0.01$)	0.06 ($P = 0.15$)	0.12 ($P = 0.04$)
KT	0.21 ($P < 0.01$)	0.04 ($P = 0.12$)	0.14 ($P = 0.03$)
F_{close}	0.46 ($P < 0.01$)	-0.03 ($P = 0.88$)	-0.04 ($P = 0.78$)

Table S2: References confirming hybridization events between species in our study and other cichlid species. We excluded references that are based on phylogenetic incongruence, because many of these cases can represent incomplete lineage sorting due to different gene coalescence that is known to be rampant in Malawi. We also limited the list to references from the primary literature and our own experiments. These constraints likely lead to underestimation of the frequency of hybridization.

Notation in Fig 2	Species A	Species B	References
a	*Labeotropheus feullebornii*	*Metriaclima zebra*	Albertson, C., Kocher, T. D. & Wainwright, P. Genetic architecture sets limits on transgressive segregation in hybrid cichlid fishes. Evolution 59, 686-690 (2005).<br>Albertson, R. C., Streelman, J. T. & Kocher, T. D. Directional selection has shaped the oral jaws of Lake Malawi cichlid fishes. Proceedings of the National Academy of Sciences of the United States of America 100, 5252-5257 (2003).<br>Danley, P. D. & Kocher, T. D. Speciation in rapidly diverging systems: lessons from Lake Malawi. Molecular Ecology 10, 1075-1086

			(2001).
b	*Cynotilapia afra*	*Metriaclima zebra*	Owen, R. et al. Major low levels of Lake Malawi and their implications for speciation rates in cichlid fishes. Proceedings of the Royal Society of London B: Biological Sciences 240, 519-553 (1990)<br>Stauffer, J.R., Jr., and E. Hert. 1992. *Pseudotropheus callainos*, a new species of mbuna (Cichlidae), with analysis of changes associated with two intra-lacustrine transplantations in Lake Malawi, Africa. Ichthyological Exploration of Freshwaters 3 (3): 253-264
c	*Metriaclima zebra*	*Metriaclima mbenji*	O'Quin CT, Drilea AC, Roberts RB, Kocher TD. 2012. A small number of genes underlie male pigmentation traits in Lake Malawi cichlid fishes. J. Exp. Zool. B 318B:199–208.
d	*Labeotropheus feullebornii*	*Tropheops* "red cheek"	Albertson, R. C., Powder, K. E., Hu, Y., Coyle, K. P., Roberts, R. B. and Parsons, K. J. (2014), Genetic basis of continuous variation in the levels and modular inheritance of pigmentation

			in cichlid fishes. Mol Ecol, 23: 5135–5150.
e	*Metriaclima zebra*	*Metriaclima benetos*	Ding B, Daugherty DW, Husemann M, Chen M, Howe AE, Danley PD (2014) Quantitative Genetic Analyses of Male Color Pattern and Female Mate Choice in a Pair of Cichlid Fishes of Lake Malawi, East Africa. PLoS ONE 9(12): e114798.
f	*Cyrtocara taeniolatus*	*Astatotilapia calliptera*	Selz, O., Lucek, K., Young, K. & Seehausen, O. Relaxed trait covariance in interspecific cichlid hybrids predicts morphological diversity in adaptive radiations. Journal of evolutionary biology 27, 11-24 (2014)
g	*Cynotilapia afra*	*Pseudotropheus elongates*	Parnell, N. F., Hulsey, C. D. & Streelman, J. T. The genetic basis of a complex functional system. Evolution 66, 3352-3366 (2012).
h	*Labidochromis* sp .	*Aulonocara* sp.	Holzman unpublished results
i	*Metriaclima callainos*	unknown cichlids	Stauffer, J. R., N. J. Bowers, T. D. Kocher, and K. R. McKaye 1996. Evidence of hybridization between

			Cynotilapia afra and *Pseudotropheus zebra* (Teleostei: Cichlidae) following an intralacustrine translocation in Lake Malawi. Copeia 1996:203–208
j	*Melanochromis aurata*	*Pseudotropheus zebra, Labeotropheus fuellbornii*	Fishelson, L. Comparison of testes structure, spermatogenesis, and spermatocytogenesis in young, aging, and hybrid cichlid fish (Cichlidae, Teleostei). Journal of morphology 256, 285-300 (2003).
